# Innovative Water-Wire Cannulation Technique for Managing Near-Complete Obstruction Due to Post-orthotopic Liver Transplant Anastomotic Strictures: A Report of Two Cases

**DOI:** 10.7759/cureus.51695

**Published:** 2024-01-05

**Authors:** Khalid Al Shamousi, Said A Al-Busafi, Ahmed Alwassief, Mohammed Al Nassar, Jawahir Lal

**Affiliations:** 1 Gastroenterology Unit, Department of Medicine, College of Medicine and Health Sciences, Sultan Qaboos University, Muscat, OMN; 2 Gastroenterology Unit, Department of Medicine, Sultan Qaboos University Hospital, Muscat, OMN; 3 Gastroenterology Unit, Department of Medicine, Sultan Qaboos University Hospital, Sultan Qaboos University, Muscat, OMN

**Keywords:** biliary strictures management, endoscopic retrograde cholangiopancreatography (ercp), orthotopic liver transplant, anastomotic strictures, water-wire cannulation technique

## Abstract

This report presents an innovative water-wire cannulation technique for managing challenging anastomotic strictures in post-orthotopic liver transplant patients, highlighting its successful application in two distinct cases. Anastomotic strictures pose a significant hurdle in hepatobiliary medicine, often complicating the course post-liver transplantation. Standard endoscopic retrograde cholangiopancreatography (ERCP) methods frequently encounter limitations in severe stricture cases, necessitating alternative approaches. The water-wire cannulation technique, introduced in this report, innovatively utilizes water injection to gently dilate the stricture, enabling successful guidewire insertion and subsequent standard endoscopic interventions. This method was effectively applied in two patients with severe anastomotic strictures, where conventional ERCP techniques were unsuccessful. The technique's effectiveness, demonstrated in these cases, offers a less invasive and potentially safer alternative to traditional options like cholangioscopy, percutaneous transhepatic cholangiography (PTC), or surgical revision, which carry higher risks and complexities. The water-wire cannulation technique's success emphasizes the need for innovative and adaptable strategies in hepatobiliary medicine, especially for managing post-transplant complications. Its potential applicability in a broader spectrum of biliary strictures warrants further exploration. Overall, this technique represents a significant advancement in the endoscopic management of complex biliary strictures, promising to enhance patient care and outcomes in hepatobiliary medicine.

## Introduction

In the realm of hepatobiliary medicine, managing anastomotic strictures post-liver transplantation remains a formidable challenge. These strictures often resist standard endoscopic retrograde cholangiopancreatography (ERCP) techniques, particularly in severe cases. This report discusses the employment of a novel water-wire cannulation technique in two distinct cases, marking a significant advancement in this field. The technique's development aligns with the ongoing need for innovative approaches in hepatobiliary medicine. Notably, anastomotic strictures, a frequent complication post-liver transplantation, have been extensively documented in medical literature as a cause of morbidity and often necessitate intervention due to their potential to disrupt biliary drainage [1]. Conventional ERCP, while a mainstay in managing biliary strictures, frequently encounters difficulties in severe post-transplant cases [2]. The water-wire cannulation technique, introduced here, represents a novel adaptation of ERCP, addressing the limitations of standard methods and offering a safer and less invasive alternative to other, more complex procedures.

## Case presentation

Case 1

**Figure 1:**
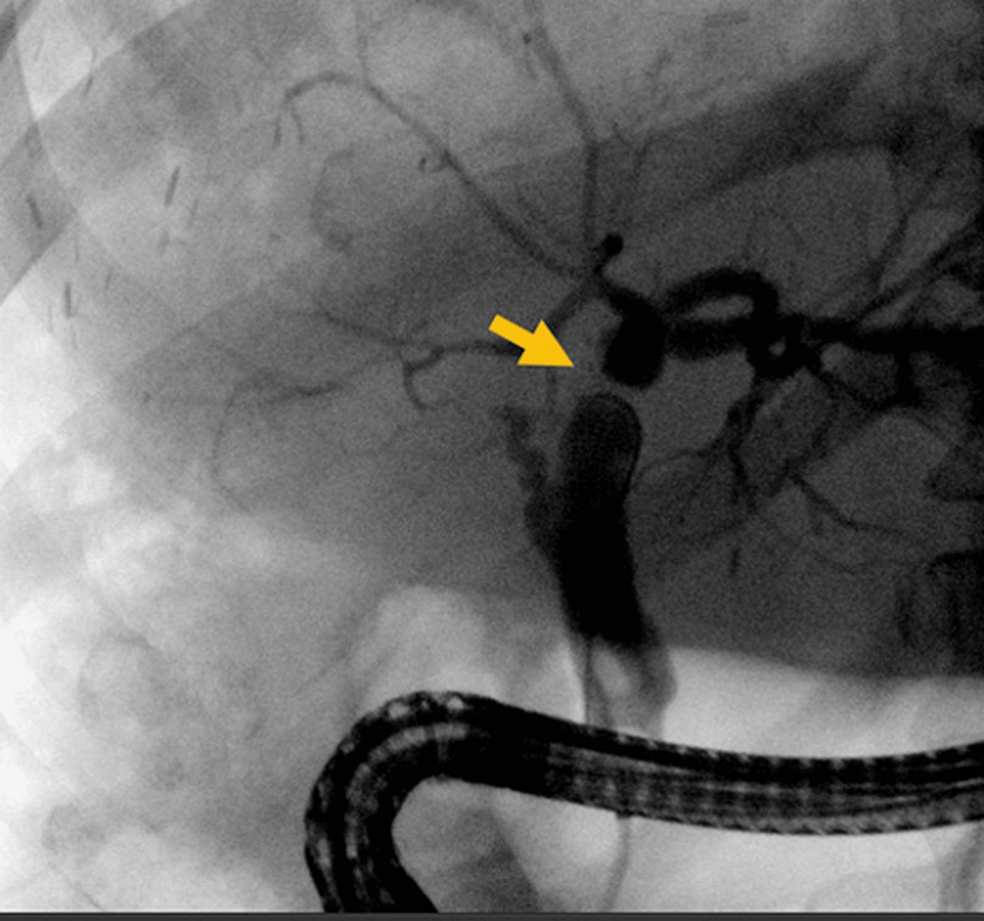
Case 1. ERCP image showing a marked anastomotic biliary stricture post-liver transplant (arrow) ERCP: endoscopic retrograde cholangiopancreatography

**Figure 2:**
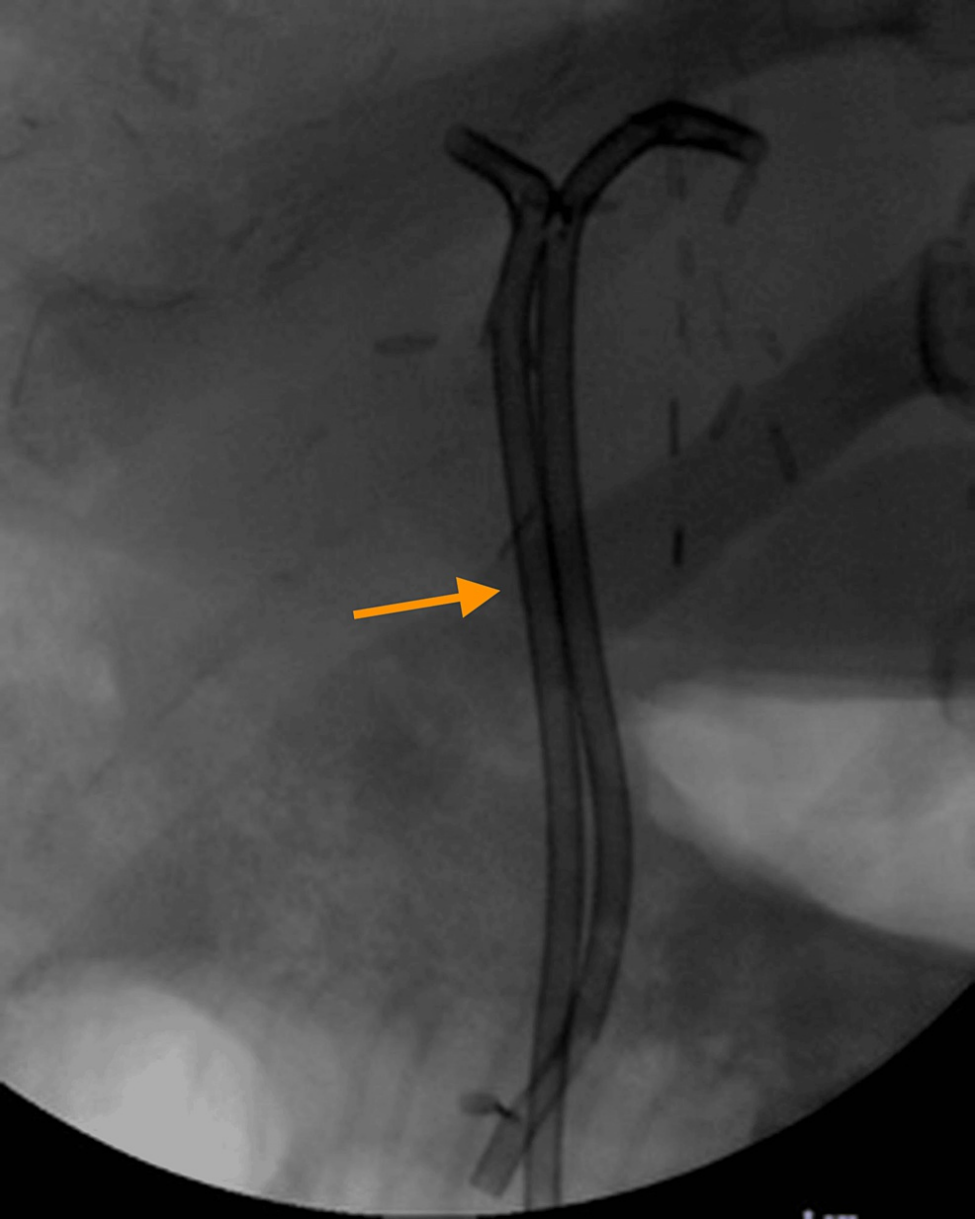
Case 1. Fluoroscopic snapshot during the ERCP highlighting the placement of two biliary stents following successful water-wire cannulation (arrow) ERCP: endoscopic retrograde cholangiopancreatography

A 58-year-old male, with diabetes mellitus (DM) and dyslipidemia (DLP), underwent a liver transplant on April 24, 2023, for biopsy-proven primary sclerosing cholangitis (PSC) and chronic cholangitis. Post-transplant, he developed an anastomotic stricture, indicated by elevated cholestatic liver enzymes. The patient underwent ERCP and stenting on June 22, 2023. During ERCP, a complete anastomotic stricture was encountered (Figure 1). Despite multiple attempts with various wires and catheters, the intrahepatic duct (IHD) could not be accessed. A novel method involving the injection of 50 ml water to dilate a potential tract was employed, successfully facilitating wire entry into the IHD. Standard therapy, including a 4F Soehendra dilator and 8 mm dilation balloon, followed (Figure 2). The laboratory results are presented in Table 1.

**Table 1:** Case 1. Liver biochemistry test prior to the ERCP ERCP: endoscopic retrograde cholangiopancreatography

	Result	Reference range
Alanine Aminotransferase	141 U/L	0 - 41
Albumin	42 g/L	35 - 52
Alkaline Phosphatase	290 U/L	40 - 129
Aspartate Aminotransferase	49 U/L	0 - 40
Total Bilirubin	6 umol/L	0 - 17

Case 2

**Figure 3:**
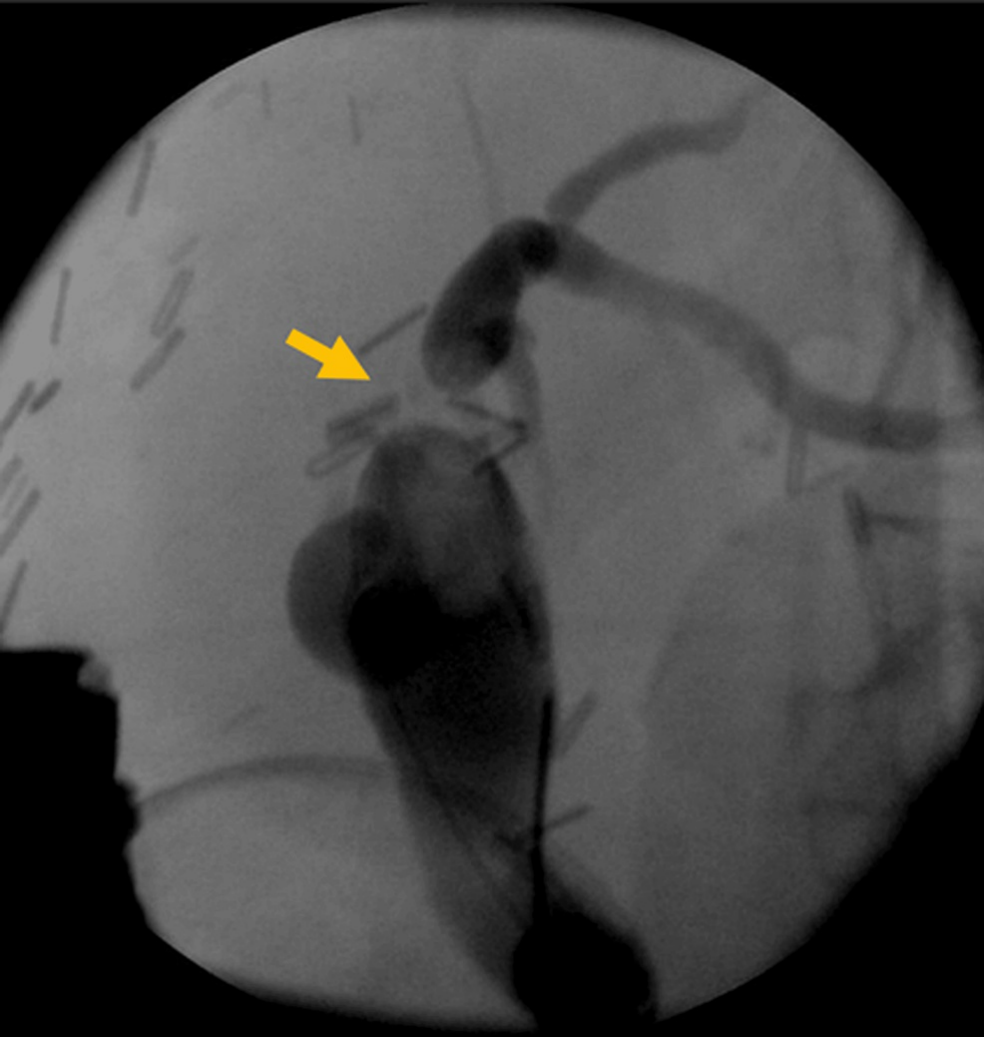
Case 2. ERCP depicting a notable anastomotic biliary stricture following liver transplantation (arrow) ERCP: endoscopic retrograde cholangiopancreatography

**Figure 4:**
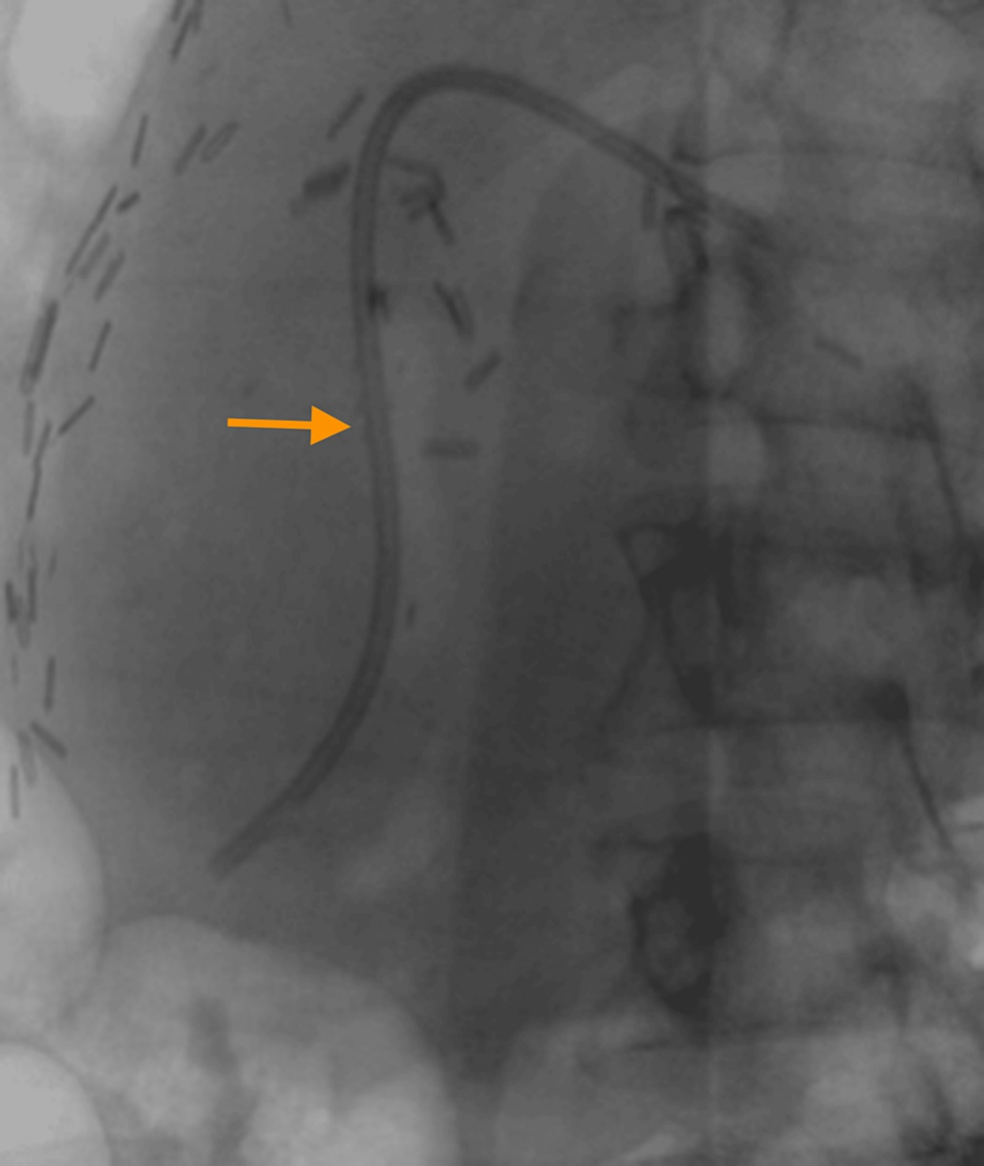
Case 2: Fluoroscopy depiction of a deeply positioned biliary stent within the hepatic duct (arrow)

A 50-year-old female underwent liver transplantation on March 8, 2022, in Turkey for non-alcoholic steatohepatitis (NASH)-related decompensated liver cirrhosis. She was also treated for adrenal insufficiency starting January 2023 (20 mg BID). An MRI indicated an anastomotic stricture (Figure 3). During ERCP, a cholangiogram revealed duct-to-duct anastomosis with dilated native and intrahepatic ducts and a tight anastomotic stricture. Similar to Case 1, the water-wire cannulation technique was employed after conventional methods failed, facilitating successful cannulation. Treatment included dilation using a Soehendra catheter and 8 mm dilation balloon, followed by placement of a 7F x 12 cm plastic stent (Figure 4). The laboratory results are presented in Table 2.

**Table 2:** Case 2. Liver biochemistry test prior to the ERCP ERCP: endoscopic retrograde cholangiopancreatography

	Result	Reference range
Alanine Aminotransferase	35 U/L	0 - 41
Albumin	37 g/L	35 - 52
Alkaline Phosphatase	167 U/L	40 - 129
Aspartate Aminotransferase	22 U/L	0 - 40
Total Bilirubin	11 umol/L	0 - 17

## Discussion

The water-wire cannulation technique (Table 3), as showcased in these cases, introduces a novel approach to the endoscopic management of challenging post-transplant anastomotic strictures. Traditional ERCP, while effective for standard biliary strictures, often fails in severe cases like those following orthotopic liver transplantation, where fibrotic changes and altered anatomy complicate cannulation [1,3]. The technique's success hinges on its ability to gently expand the stricture using water injection, creating a more favorable environment for guidewire insertion. This method may circumvent the need for more invasive procedures like percutaneous transhepatic cholangiography or surgical revision, which carry higher risks and longer recovery times.

**Table 3:** Sequential steps of the ERCP using the water-wire cannulation technique ERCP: endoscopic retrograde cholangiopancreatography

Step Number	Procedure Phase	Description of Step
1	Initial assessment and setup	Review patient history and imaging, sedate patient, position for ERCP, and prepare endoscopic equipment.
2	Duodenoscope Insertion and papillary access	Insert the duodenoscope into the duodenum and navigate to the major papilla into the common bile duct.
3	Stricture identification	Locate the stricture site visually or using fluoroscopy; assess its severity and characteristics.
4	Water injection procedure	If standard cannulation fails, inject 50 ml of sterile water into the stricture area to expand it.
5	Guidewire insertion	Quickly re-introduce the guidewire after water injection to utilize the temporary dilated path.
6	Wire navigation and stricture dilation	Pass the guidewire through the stricture, then dilate the stricture using a dilating catheter or balloon. Olive oil can be used to lubricate the dilation balloon to reduce the friction at the stricture and ease entry.
7	Therapeutic interventions	Perform necessary interventions such as further dilation, stent placement, or stone removal. Use fluoroscopy for guidance and confirmation.
8	Completion and withdrawal	Carefully withdraw instruments after completion, and inspect the area to ensure no complications have occurred.

In contrast to the innovative water-wire cannulation technique, traditional options for managing post-transplant anastomotic strictures include cholangioscopy, percutaneous transhepatic cholangiography (PTC), and surgical revision [2,4-6]. Cholangioscopy offers direct visualization of the biliary tree, allowing for targeted therapy but is limited by its technical complexity and the need for specialized equipment [7]. Percutaneous transhepatic cholangiography, on the other hand, provides an alternative route for biliary access and intervention, especially in patients with biliary enteric anastomotic strictures [5]. While effective, PTC is more invasive and associated with a higher risk of complications such as bleeding, bile leaks, and infection. Surgical revision, the most invasive option, is usually reserved for cases where endoscopic and percutaneous approaches fail. It involves re-establishing biliary drainage through reconstructive surgery, carrying the inherent risks of major surgery, including longer recovery times and higher morbidity. While effective in many cases, these traditional methods underscore the need for less invasive and safer techniques like water-wire cannulation in managing complex post-transplant biliary strictures.

The successful application of the water-wire cannulation techniques in these cases also highlights the importance of innovation and adaptability in hepatobiliary medicine. Anastomotic strictures pose a significant risk of morbidity and mortality due to complications like cholangitis and liver failure. This technique improves patient outcomes by offering a less invasive yet effective solution.

Furthermore, this technique's simplicity and safety profile suggest potential applicability in other complex biliary strictures beyond the post-transplant setting. Its ability to negotiate tight strictures without excessive force minimizes the risk of perforation and other ERCP-related complications. Future studies could explore its efficacy and safety in a broader range of biliary obstruction scenarios.

## Conclusions

In conclusion, the innovative water-wire cannulation technique detailed in this report heralds a new chapter in the endoscopic management of anastomotic strictures post-orthotopic liver transplantation. The success of this method in two complex cases where traditional ERCP failed demonstrates its potential as a less invasive and safer alternative, which may revolutionize the approach towards these challenging conditions. The technique's ability to delicately dilate strictures and facilitate guidewire insertion without the need for more invasive procedures suggests its applicability could extend beyond the scope of current practice. It mitigates the risk associated with traditional methods and offers a promising solution to improve patient outcomes.

Future research should aim to substantiate these preliminary findings through larger studies to confirm the technique’s efficacy and safety profile. The potential for this technique to become a standard part of the hepatobiliary therapeutic arsenal is immense, offering hope for better management of post-transplant biliary complications. The water-wire cannulation technique is more than a procedural innovation; it is a testament to the importance of adaptability and creative problem-solving in clinical practice. Its success underscores the significance of continuous innovation in the field of hepatobiliary medicine, with the ultimate goal of providing patient-centered care that is both effective and minimally invasive.
